# Experiences and Acceptance of Community-Based Mobile Health Services Among People in Underserved Rural Areas of Korea: Mixed Methods Study

**DOI:** 10.2196/91368

**Published:** 2026-06-22

**Authors:** Dong-soo Shin, Jin Soon Kim, Kunhee Park, Minseok Pyo, Sung Eun Li, Minjin Kim, Jungae Lee, Byeong Yeob Oh, Yong-jun Choi, Ben Gerber, Adrian Zai, Jeroan Allison

**Affiliations:** 1 School of Nursing & Research Institute of Nursing Science, Hallym University, , Chuncheon, Gangwon-do, Republic of Korea; 2 Global Frontier Research Center, Hallym University, , Chuncheon, Gangwon-do, Republic of Korea; 3 Pyeongchang County Health and Medical Center, Pyeongchang-eup, Republic of Korea; 4 Korea Health Promotion Institute, Seoul, Seoul, Republic of Korea; 5 University of Cincinnati, College of Nurisng, Cincinnati, OH, United States; 6 Department of Population and Quantitative Health Sciences, University of Massachusetts Chan Medical School, Worcester, MA, United States; 7 Care Square, Seoul, Republic of Korea; 8 Department of Social and Preventive Medicine & Institute of Social Medicine, College of Medicine, Hallym University, 1 Hallymdaehak-gil ChunCheon Gangwondo, 2515, Chunceon, Gangwon-do, 24252, Republic of Korea, 82 1096803483, 82 33-248-2734

**Keywords:** mobile health, rural health, underserved populations, chronic disease, Unified Theory of Acceptance and Use of Technology 2, UTAUT2, self-management

## Abstract

**Background:**

Community-based mobile health (mHealth) services are increasingly used to support chronic disease management in underserved rural populations facing workforce shortages, geographic isolation, and rapid aging. South Korea entered a super-aged society in December 2024, intensifying pressures in rural regions where multiple mHealth programs are embedded within primary care and public health systems. However, evidence on sustained use in real-world settings remains limited.

**Objective:**

This study aimed to explore user experiences and acceptance of community-based mHealth services in an underserved rural area of South Korea and identify facilitators and barriers to sustained engagement, using the Unified Theory of Acceptance and Use of Technology 2 (UTAUT2).

**Methods:**

A convergent mixed methods design was used, with qualitative and quantitative data collected in parallel, analyzed separately, and integrated at the interpretation stage. Overall, 24 participants with ≥6 months of experience using 1 of 4 publicly funded mHealth services in Pyeongchang County, Gangwon State, were purposively recruited. Semistructured interviews guided by the UTAUT2 were analyzed using directed content analysis, combining deductive and inductive coding. Structured questionnaires assessing usability and behavioral intention were analyzed using descriptive statistics. Findings were integrated through joint interpretation.

**Results:**

Participants had a mean age of 71.3 (SD 9.2) years, and 70.8% (17/24) were female; hypertension (18/24, 75%) and hyperlipidemia (15/24, 58.3%) were the most common. Perceived difficulty was low (mean 2.54, SD 2.06, on a 0‐10 scale), intention for continued use was high (23/24, 95.8%), and recommendation intention was unanimous (24/24, 100%). Willingness to pay was reported by 79.2% (19/24), most commonly KRW 1000‐5000 (US $1-3) per month. Qualitative findings identified performance expectancy, social influence, facilitating conditions, and habit as the most salient determinants of sustained use. Real-time monitoring enhanced health awareness, motivated dietary modification, and increased physical activity. Public health center nurses served as human-in-the-loop facilitators, providing continuous training, troubleshooting, and emotional support, while family and peers reinforced engagement. Habit formation emerged as a central mechanism, with 91.7% (22/24) integrating mHealth use into routines anchored to waking, exercise, and bedtime. Effort expectancy barriers among older participants were mitigated through nurse-led training, and hedonic motivation was driven by intrinsic satisfaction and peer interaction. Integrated analysis showed convergence for ease of use and behavioral intention, and partial divergence for willingness to pay.

**Conclusions:**

Community-based mHealth services were successfully integrated into daily life and supported chronic disease self-management among older adults in an underserved rural setting. Sustained engagement was driven by perceived health benefits, continuous human support, and habit formation rather than technology features alone, underscoring the importance of relationship-centered, human-in-the-loop implementation models. Strengthening intuitive design, hands-on onboarding, multidisciplinary primary care teams, and stable financing will be essential for equitable digital health adoption in rural and aging communities.

## Introduction

Chronic disease management is a complex, lifelong process that requires sustained collaboration among patients, family members, neighbors, and multidisciplinary primary care teams, including physicians, nurses, pharmacists, and other allied health professionals [1]. Trust-based communication and continuous encouragement are essential to maintaining self-management efforts and preventing disengagement over time [2]. To enhance patient engagement, remote patient monitoring and other digital health tools have increasingly been integrated into chronic care models. Evidence suggests that when these technologies are combined with nurse-led or team-based human-in-the-loop approaches, they lead to greater improvements in clinical outcomes and patient-reported experiences than technical solutions alone [3-5].

Despite these advances, significant disparities in access to and quality of chronic disease management persist in rural and underserved areas [6]. In rural areas, these disparities are further exacerbated by interconnected structural factors, including geographic isolation, limited health care infrastructure, shortages of health care professionals, and transportation barriers. These constraints reduce access to timely and continuous care and contribute to delayed detection of health deterioration and poorer chronic disease outcomes [7]. In this context, mobile health (mHealth) technologies have been proposed to bridge these systemic gaps by enabling remote monitoring, facilitating patient-provider communication, and supporting self-management in daily life.

In December of 2025, South Korea faces a super-aged society by 8 years since 2017, when the 65 years or older comprised 14% of the population [8]. This rapid population aging places substantial pressure on rural communities, where limited health care infrastructure and workforce shortages hinder timely adaptation, making it a critical policy issue in the Korean health and social care system. In addition, rural population decline has been driven by sustained out-migration of younger populations to urban areas, combined with low fertility and demographic aging. These changes have weakened local economies and reduced the availability of essential services and infrastructure, further exacerbating shortages of health care and community resources in rural regions [9].

Pyeongchang County, located in Gangwon State, has a markedly aged population, with 37.2% of residents aged 65 years or older [10]. The region faces notable health care access barriers, including transportation inconvenience, limited availability of medical services, and unmet health care needs, particularly among older adults in geographically dispersed areas [11]. In addition, the county demonstrates a substantial burden of chronic conditions among older adults, with hypertension affecting approximately 64% of individuals aged 65 years or older and diabetes affecting 29.3%, based on the 2023 Korea Community Health Survey [12]. These characteristics make Pyeongchang an appropriate and policy-relevant setting for applying community-based mHealth services aimed at supporting older adults and improving chronic disease management in underserved rural contexts. These include programs, such as Mobile Healthcare Service, Gangwon Health Up, Value Health Services, and artificial intelligence (AI)/IoT Senior Health Management Service, which support real-time monitoring of blood pressure, blood glucose, and physical activity, along with tailored feedback and coaching [13-16]. Rather than being isolated pilot interventions, these programs collectively represent an emerging regional ecosystem of digitally supported chronic disease management embedded within primary care and public health systems.

While such initiatives demonstrate the potential of mHealth to enhance access and continuity of care, their successful implementation in underserved rural populations remains challenging. Rural residents often face multiple barriers to digital health adoption, including low digital literacy, limited access to devices or stable connectivity, and social isolation [17-19]. In addition, perceptions of value, including whether the benefits of digital health services justify potential costs, may influence acceptance and sustained use, particularly in resource-constrained settings. Understanding how individuals in underserved rural areas experience, perceive, and integrate mHealth services into their daily lives is therefore critical for designing equitable and sustainable digital health interventions.

The Unified Theory of Acceptance and Use of Technology 2 (UTAUT2) provides a comprehensive framework for examining determinants of technology adoption and sustained use in consumer health contexts [20]. UTAUT2 extends the original model by incorporating 7 constructs particularly relevant to everyday technology use. Performance expectancy refers to the extent to which individuals believe that using a digital health solution will improve health outcomes or enhance self-management effectiveness. Effort expectancy captures perceived ease of learning and using the technology. Social influence reflects the degree to which individuals perceive encouragement or expectations from family members, peers, and health care professionals to use the technology. Facilitating conditions denote the availability of organizational and technical infrastructure, including training, technical support, and necessary resources. Hedonic motivation represents the enjoyment and satisfaction derived from technology use. Habit refers to the extent to which technology use becomes automatic and embedded in daily routines through repeated practice and is a critical determinant of long-term engagement. Price value reflects users’ evaluation of the benefits of using a technology relative to its monetary cost, influencing behavioral intention and continued use [20].

Although UTAUT2 has been widely applied to understand the acceptance of digital health technologies among older adults and individuals with chronic conditions, relatively few studies have examined its application in community-based mHealth services embedded within primary care systems serving underserved rural populations [21-24]. In particular, limited evidence exists on how structural constraints, human support systems, and contextual factors interact to shape technology acceptance and sustained engagement in real-world rural settings.

Therefore, this study aims to explore the lived experiences and acceptance of community-based mHealth services among residents in an underserved rural area of South Korea. Using a convergent mixed methods design guided by the UTAUT2 framework, this study examines how mHealth services are experienced in everyday life, identifies key facilitators and barriers to sustained use, and explores how community-based public health systems support digital health adoption and chronic disease self-management. The findings aim to inform actionable strategies for improving digital health delivery and advancing digital health equity in underserved rural communities.

## Methods

### Study Design

The study was informed by a pragmatic research paradigm, integrating theory-driven (UTAUT2-based) and data-driven approaches to understand real-world user experiences and implementation processes. A convergent mixed methods design was used, in which qualitative and quantitative data were collected in parallel, analyzed separately, and subsequently integrated at the interpretation stage to provide a comprehensive understanding of participants’ experiences and acceptance factors [25].

### Setting and Context

The study was conducted in Pyeongchang County in South Korea. The county is characterized by geographic dispersion with limited public transportation infrastructure. Residents must travel 40‐80 kilometers to access hospital services. At the time of this study, 4 major mHealth services were implemented across Gangwon State (Table 1). Each service leverages a mobile platform and is connected to biometric devices to support chronic disease management. Three services, Gangwon Health Up, Value Health, and AI/IoT Senior Health Management Service, primarily target adults and older adults diagnosed with hypertension or diabetes, whereas the mobile health care service focuses on adults with risk factors for metabolic syndrome. Smartphone apps, Bluetooth-enabled devices, and algorithm-based feedback systems provide support in tracking blood pressure, blood glucose, physical activity, dietary behaviors, and other health indicators. Periodic assessments by health care staff, mostly nurses, are conducted through public health centers using digital reporting systems. Together, these 4 interconnected services constitute Gangwon State’s digital health ecosystem, offering technology-assisted self-management, individualized monitoring, and coordinated public health support for chronic disease prevention and control.

**Table 1.** Mobile health services in research sites.

Service, start year	Lead agencies	Digital tools	Monitoring indicators	Target population	Evaluation
Mobile Healthcare service, 2016	Ministry of Health; KHPI^a^; KOSIS^b^	Chaeum Health app; Bluetooth devices	BP^c^, glucose, lipids, waist circumference, physical activity, diet	Adults ≥19 years with metabolic syndrome risk factors	Baseline, 3-month, 6-month biometric and lifestyle assessment
Gangwon Health Up, 2022	Gangwon Provincial Government	Health Up app; smart band	Steps, lifestyle logs, dietary photo analysis, BP or glucose, AI CVD^d^-risk	Local adults with hypertension or diabetes or seeking lifestyle improvement	Initial + continuous monitoring + final evaluation
Value Health Service, 2024	Seoul National University and its consortium	Value Health web and app; Bluetooth BP or glucose	Lifestyle factors, BP, glucose	Individual users, primarily with hypertension or diabetes	Pre–post 6‐9 month evaluation
AI/IoT Senior Health Management Service, 2020	Ministry of Health; KHPI	Today’s Health app; AI speaker; Bluetooth BP, glucose, weight, activity devices	BP, glucose, weight, physical activity, medication, lifestyle	Adults ≥65 years diagnosed with hypertension or diabetes, or needing lifestyle management	Pre–post 6-month evaluation

a KHPI: Korea Health Promotion Institute.

b KOSIS: Korean Statistical Information Service.

c BP: blood pressure.

d AI CVD: artificial intelligence-based cardiovascular risk.

### Participants and Recruitment

Purposive sampling was used to recruit participants through health care staff at public health centers. Inclusion criteria were as follows: (1) at least 6 months of experience using mHealth services, (2) ability to communicate in Korean, and (3) willingness to provide informed consent. Participants were excluded if they used the digital health application less than one-third of weekdays (fewer than 6 days per month), as this level of engagement was considered insufficient to provide meaningful insights into their experiences with the mHealth services.

### Data Collection

#### Qualitative Data

In-depth semistructured interviews were conducted by DS and JS between December 1‐5, 2025. The interview guide was developed based on the UTAUT2 theoretical constructs, with the exception of price value, which was assessed quantitatively (Table 2). The guides explored participants’ perceptions of using mobile devices for monitoring blood pressure and blood glucose and whether they believed these activities could improve their health outcomes. Participants were asked about the effort required to use the technology, sources of support (including where support was obtained and from whom), perceived ease or difficulty of use, enjoyment, and the extent to which the technology had been integrated into their daily routines. Each interview lasted approximately 30‐60 minutes and was audio-recorded with participants’ informed consent. All recordings were transcribed verbatim in Korean, and detailed field notes were maintained to document contextual observations and nonverbal cues observed during the interviews.

**Table 2.** Unified Theory of Acceptance and Use of Technology 2–based semistructured interview guide.

Domain	Questions
Performance expectancy	Q1. While using this service (app or device), did you experience any changes in blood pressure or blood glucose management, dietary habits, physical activity, or hospital visits? Q2. In what ways did the service help you manage your health? Please describe in detail.
Effort expectancy	Q1. Did you experience any difficulties when you first started using the device? Q2. What aspects of learning to use the app or device were difficult or easy? Q3. What efforts did you make to become familiar with it? (eg, assistance from family members, support from staff, and repeated practice)
Social influence	Q1. How did your family members, neighbors, or friends react to your use of the app or device? Q2. Did their reactions influence your willingness to use the service?
Facilitating conditions	Q1. When you needed help during use, did you receive sufficient support (eg, education, counseling, and troubleshooting)? Q2. How did you handle device malfunctions, errors, or connectivity issues? Q3. Did you have any concerns or discomfort regarding data privacy or sharing your results with family members or health care providers?
Hedonic motivation	Q1. Were there features such as encouragement messages, rewards, or challenge-based activities included in the service? Q2. Which of these features were motivating for you?
Habit	Q1. When and in what situations did you mainly use the app or device? (eg, immediately after waking up, after taking medication, after exercise) Q2. Did you have any personal strategies to maintain consistent use (eg, setting alarms and support from family members)?

#### Quantitative Data

A structured questionnaire was administered to all participants following completion of the qualitative interviews. The questionnaire collected demographic and health-related characteristics, including age, gender, prevalence of chronic conditions, duration of service use, and preferences for digital health outputs (eg, numeric values, graphical trends, or other formats). Perceived difficulty of device use was assessed on a scale from 0 (very easy) to 10 (very difficult). Intention for long-term use and recommendation of the app to others were assessed using yes or no questions. Because the mHealth services were provided free of charge at the time of the study, price value was assessed based on participants’ willingness to pay if the service were to become fee-based. These items were included to facilitate the expression of participants’ perspectives, particularly in cases where individuals may have difficulty articulating their opinions explicitly [26].

### Data Analysis

#### Qualitative Analysis

Transcripts were analyzed using directed content analysis [25], with deductive coding guided by UTAUT2 constructs and additional inductive coding to identify new codes not captured by the predefined constructs. ChatGPT (OpenAI) was used as an assistive analytic tool to support deductive content analysis by generating preliminary codes and summaries aligned with the UTAUT2 framework. Consistent with prior evidence on the reliability of large language models in qualitative content analysis, all AI-generated outputs were independently reviewed, refined, and validated by researchers to ensure analytic rigor and credibility [27]. Two researchers (DS and JS) independently extracted codes and narratives and coded the transcripts. Discrepancies were discussed until consensus was reached. KH and YJ reviewed and confirmed the coding framework.

From the 6 UTAUT2 constructs, 23 initial codes were identified; after iterative refinement, 19 codes were retained, with 3‐4 key codes per construct. For performance expectancy, 3 codes were identified: data-driven health awareness, behavior activation, and perceived health benefits. Preventive health orientation was initially identified but later integrated into perceived health benefits. For social influence, 3 codes were identified: family support, peer influence, and provider recommendation. Social accountability was initially identified but later subsumed under family and peer influence due to conceptual overlap with interpersonal social dynamics. Facilitating conditions comprised technical support availability, supported learning, continuous guidance, and a human-mediated system. Habit was represented by routine embedding, automaticity, and cue-triggered action. Effort expectancy included 3 codes: initial usability barriers, supported adaptation, and intuitive and accessible design. Hedonic motivation included 3 codes: enjoyment of monitoring, achievement satisfaction, and intrinsic motivation. Gamification was integrated into achievement satisfaction, and extrinsic reward influence was excluded due to low relevance (Table S1 in Multimedia Appendix 1).

#### Quantitative Analysis

Descriptive statistics, including mean, SDs, and frequencies, were calculated using Microsoft Excel. Integration of qualitative and quantitative findings was accomplished during the interpretation stage to generate comprehensive insights.

Rigor and Trustworthiness Methodological rigor and trustworthiness were ensured through 4 strategies. Credibility was enhanced through immediate debriefing sessions conducted after each interview session, allowing researchers (DS and JS) to capture key meanings and refine interpretations in real time. Dependability was supported by applying a consistent coding framework based on UTAUT2 constructs across all transcripts, ensuring analytic coherence throughout the study. Confirmability was strengthened by having 2 researchers (DS and JS) independently analyze the data and resolve discrepancies through discussion and consensus, thereby minimizing individual bias. Transferability was addressed by providing thick descriptions of the study setting, participant characteristics, and the broader rural context, enabling readers to assess the applicability of the findings to similar populations and settings.

### Ethical Considerations

This study was approved by the Institutional Review Board of Hallym University (HIRB-2025‐129). All participants provided written informed consent prior to participation. To ensure privacy and confidentiality, all participant names were removed from the transcripts and responses and replaced with sequential identification numbers. All data were stored securely and accessible only to the research team. As a token of appreciation, participants were provided with gift certificates.

## Results

### Participant Characteristics

Characteristics of the study participants are presented in Table 2. The study included 24 participants, with 6 individuals enrolled in each mHealth service. The mean age was 71.3 (SD 9.2) years, and most participants were female (17/24, 70.8%). Participants commonly had multiple chronic conditions, with hypertension being the most prevalent (18/24, 75%), followed by hyperlipidemia (14/24, 58.3%), diabetes (10/24, 41.7%), and osteoarthritis (6/24, 25%). In terms of service engagement, 33.3% (8/24) of participants had used the services for 6‐12 months, 41.7% (10/24) for 1‐3 years, and 25% (6/24) for more than 3 years.

### Quantitative Findings Related to Technology Use

Quantitative findings related to technology use are summarized in Table 3. Participants reported a low perceived difficulty score, with a mean of 2.54 (SD 2.06) on a 0‐10 scale, where higher scores indicate greater perceived difficulty. This suggests that, overall, participants perceived the mHealth services as relatively easy to use.

**Table 3.** Participants’ characteristics (n=24).

Characteristic	Values
Age (years), mean (SD)	71.3 (9.2)
Gender, n (%)	
Women	17 (70.8)
Men	7 (29.2)
Chronic conditions, n (%)	
Hypertension	18 (75)
Diabetes	10 (41.7)
Hyperlipidemia	14 (58.3)
Osteoarthritis	6 (25)
Service usage duration, n (%)	
6‐12 months	8 (33.3)
1‐3 years	10 (41.7)
>3 years	6 (25)
Digital health service used, n (%)	
Mobile Healthcare	6 (25)
Gangwon Health Up	6 (25)
Value Health	6 (25)
Today’s Health	6 (25)

Most participants expressed strong behavioral intention toward continued use. Specifically, 95.8% (23/24) indicated that they intended to continue using the services long term, while only 1 participant reported otherwise. Recommendation intention was uniformly high, with all participants indicating that they would recommend the service to others.

Willingness to pay for continued use was less consistent compared to other indicators. While the majority of participants (19/24, 79.2%) reported willingness to pay, a subset (5/24, 20.8%) indicated that they would prefer to use the service only if it remained free. Among those willing to pay, the most commonly reported acceptable monthly cost ranged between KRW 1000 and 5000 (US $1-3) (11/24, 45.9%), followed by KRW 6000‐10,000 (US $4-7) (6/24, 25%) and KRW 10,000‐20,000 (US $7-13) (2/24, 8.3%) (Table 4).

**Table 4.** Quantitative findings related to technology use (n=24).

Variables	Values
Perceived ease of use (0‐10), mean (SD)	2.54 (2.06)
Intention for long-term use, n (%)	
Yes	23 (96.8)
No	1 (4.2)
Recommendation intention, n (%)	
Yes	24 (100)
Willingness to pay, (KRW), n (%)	
Free of charge	5 (20.8)
1000~5000^a^	11 (45.9)
6000~10,000^b^	6 (25)
10,000~20,000^c^	2 (8.3)

a US $1-3.

b US $4-7.

c US $7-13.

Across the UTAUT2 constructs, performance expectancy, social influence, facilitating conditions, and habit emerged as the most salient drivers of acceptance and continued use. Effort expectancy and hedonic motivation were present but less prominent, likely because sustained nurse support reduced usability barriers over time, and enjoyment emerged primarily after routines were established.

### Performance Expectancy

#### Real-Time Monitoring as a Motivator for Self-Management

Participants strongly believed that using digital health devices motivated them to engage in self-management through real-time monitoring of their changes. This increased engagement in chronic illness management and prevention activities, which they perceived as contributing to improved health outcomes. Perceived benefits included better blood pressure control, early detection of abnormal glucose levels, increased physical activity, and weight management.

This device helps me check my numbers every day. Before, I only checked at the hospital every three months. Now I can see immediately how my food choices affect my blood sugar.

[Female, 77 years old]

I started eating less carbohydrates and more vegetables after seeing my daily blood pressure. Walking around 7000‐8000 steps daily became my habit. The data motivated me to keep going.

[Female, 73 years old]

Before using this service, I didn’t know I was at risk for diabetes. Now I caught it early (pre-diabetes stage) and changed my diet. The doctor said I avoided progressing to diabetes because of daily monitoring.

[Female, 73 years old]

However, participants expressed emotional discomfort with the feedback system used in the mHealth service. When their blood pressure readings fell outside the normal range, one program displayed a “Caution” alert. Several participants reported feeling discouraged or unfairly reprimanded despite their efforts to engage in self-management and requested that the message be revised to more supportive terms such as “Let’s improve together” or “Adjustment recommended.” In contrast, another program displayed red-colored alerts only to the nurses, not to the patients, allowing nurses to contact participants directly and identify the underlying reasons for the abnormal readings without causing unnecessary anxiety for participants.

### Social Influence

#### Motivation Through Family, Peers, and Health Care Providers

Social influence from family members, neighbors, and health care providers played an important role in participants’ adoption and continued use of mHealth services. Family members often acted as motivators, reminding participants to use the devices and engaging in shared health activities that strengthened mutual accountability. Peer influence was similarly powerful, as seeing friends use the devices or receiving encouragement fostered motivation and pride in maintaining healthy routines. Recommendations from health care providers also influenced participants’ decisions to join the service. As one participant explained, “The doctor recommended this service, and I trusted his advice, so I joined.” Together, these interpersonal influences reinforced participants’ engagement and contributed to sustained use of digital health devices.

### Facilitating Conditions

#### Nurses as a Human-in-the-Loop

The research was conducted in government-operated public health centers, where nurses assumed central coordinating roles. Beyond routine clinical duties, they offered technical troubleshooting, device training, and ongoing maintenance. Consequently, participants depended extensively on these public health center nurses to sustain their digital health use.

Whenever my device doesn’t connect, I call the health center. The nurse fixes it immediately or I bring it in. Without this support, I would have given up.

[Female, 69 years old]

The health coordinator who is a nurse visits the exercise group weekly. She checks the devices and answers questions. This regular contact keeps me engaged.

[Female, 80 years old]

Training for device setup and maintenance was brief for most users, but it was repeated across several months for the oldest participants and those struggling with digital literacy. Many noted that hands-on, step-by-step demonstrations were more effective than written guidance.

### Habit

#### Became a Part of My Daily Routine

Habit formation emerged as the strongest predictor of sustained mHealth service use among participants. The vast majority of participants (22/24, 91.7%) successfully integrated digital health monitoring into their established daily routines, demonstrating deep behavioral incorporation. Morning routines typically begin with waking up, immediately checking the device, measuring blood pressure, and reviewing data summary from the previous day. After exercise sessions, participants would check their step count and heart rate to assess their physical activity. Before bed, they would review their daily summary to reflect on the day’s health metrics and ensure their device was charged for the next day’s use.

I wear my device all the time, even when sleeping. I only remove it for showering. Without it, I feel like something is missing.

[Female, 80 years old]

Checking my blood pressure every morning became automatic, like brushing my teeth. I don’t even think about it anymore.

[Female, 73 years old]

When the program paused for a year, I felt lost. I bought a commercial smartwatch to continue tracking. When the program restarted, I immediately rejoined.

[Female, 73 years old]

Several factors facilitated successful habit formation among participants. Consistent daily timing allowed health monitoring activities to become automatic components of established routines, reducing the cognitive effort required to remember to use devices. The wearable design of devices ensured they were always accessible and physically present, serving as constant tangible reminders. Visual reminders delivered through app notifications prompted users at strategic times throughout the day. Social accountability mechanisms, including peer monitoring and nurse check-ins, created external motivation structures that reinforced individual commitment to consistent device use.

### Effort Expectancy

#### Easy to Use

Ease of use varied considerably across participants and was strongly influenced by age, prior digital experience, and cognitive function. Most participants reported that the technology was generally accessible and intuitive; however, older participants encountered more challenges during the initial learning phase and required a longer adaptation period, often more than 6 months, for sustained use. Users of the Mobile Healthcare service, who had metabolic syndrome and were relatively younger than the other groups, reported fewer usability challenges overall. Across all sites, nurses played a pivotal role in helping participants become accustomed to the devices by providing repeated guidance and reassurance.

It was easy for me. I only asked the nurse a few times. After that, I could do everything myself.

[Female, 63 years old]

At first, it was confusing. I rated difficulty as 6/10. But the nurse helped me many times. Now it feels automatic.

[Female, 80 years old]

#### Simple and Visual Display

When discussing their experiences with device usability, participants emphasized the critical importance of intuitive design principles. They expressed strong preference for one-touch operation over multistep processes, which could easily become confusing or overwhelming. Visual icons proved far more effective than text-based instructions for conveying functions and options. Participants also highlighted that large buttons and clear displays were essential design features for aging eyes, enabling them to interact with devices confidently and accurately without straining to read small text or locate tiny controls.

For older adults, it has to be easy. Simple. Something they can understand at a glance

[Female, 80 y old]

### Hedonic Motivation

#### Enjoyment, Achievement, and Playfulness in Daily Self-Care

Participants reported deriving considerable enjoyment from multiple aspects of mHealth service use. Achieving daily goals for steps or blood pressure and visualizing their progress over time provided a sense of accomplishment and satisfaction. Receiving positive feedback and encouragement from the app, health care providers, or peers reinforced their motivation to continue. Friendly competition with peers added a social and playful dimension to health management activities. Small rewards such as gifts or recognition ceremonies further enhanced their enjoyment and sense of validation for their efforts.

I enjoy seeing my blood pressure each morning. It’s like a game—I try to maintain my blood pressure within normal range.

[Female, 69 years old]

When I reached 10,000 steps, the app celebrated with me. I felt proud. Small rewards like protein drinks motivate me to keep going.”

[Female, 80 years old]

However, some participants noted that extrinsic rewards (gifts) were less important than intrinsic motivation (health improvement).

Gifts are nice, but I use this service because I want to stay healthy, not for prizes. My health is my greatest reward.

[Female, 73 years old]

#### Integration of Qualitative and Quantitative Findings

Integrated analysis demonstrated overall convergence between quantitative and qualitative findings Table 5. Perceived ease of use was generally low (mean 2.54, SD 2.06); however, qualitative findings indicated that ease of use reflected a process of supported adaptation through repeated training and ongoing support rather than inherent system simplicity. A subgroup (5/24, 20.8%) reported higher difficulty, primarily related to initial learning barriers and device issues, which were mitigated by external support. Most participants reported intention for continued use (23/24, 95.8%), which was explained by perceived health benefits (performance expectancy), habit formation, and facilitating conditions identified in qualitative findings. In contrast, recommendation intention (24/24, 100%) was primarily associated with social influence, reflecting encouragement from family, peers, and health care providers, as well as positive user experiences. Willingness to pay showed partial divergence. Although most participants were willing to pay (19/24, 79.2%), some preferred the service to remain free, reflecting variability in perceived value influenced by individual experiences and contextual expectations.

**Table 5.** Integrated interpretation of quantitative and qualitative findings across usability, behavioral intention, and price value (n=24).

Quantitative variable	Quantitative result	Linked UTAUT2^a^ constructs	Interpretation type and description
Perceived ease of use (0‐10)	Mean 2.54 (SD 2.06); higher difficulty (≥5) 5/24 (20.8%)	Effort expectancy; Facilitating conditions	Complementarity: overall perceived difficulty was low; however, qualitative findings indicated that ease of use resulted from repeated training and ongoing support, reflecting a process of supported adaptation rather than inherent simplicity Complementarity: a subgroup experienced usability challenges related to device errors and initial learning barriers. These difficulties were mitigated through external support (nurses, family), allowing continued use despite higher perceived difficulty.
Intention for long-term use	Yes: 23/24 (95.8%)	Performance expectancy; Habit; Facilitating conditions	Convergence: most participants reported intention for continued use, explained by perceived health benefits, routine integration, and sustained human support.
Recommendation intention	Yes: 24/24 (100%)	Social influence; Performance expectancy	Convergence: universal recommendation intention aligned with qualitative findings on social encouragement and positive health experiences, indicating strong social diffusion.
Willingness to pay (KRW)	Yes: 19/24 (79.2%); no: 5/24 (20.8%)	Price value; Performance expectancy; Facilitating conditions	Partial divergence: while most participants were willing to pay a modest fee, some perceived limited added value, increased self-efficacy, device issues, and expectations for public support, indicating that price value is dynamic and context-dependent.

a UTAUT 2: Unified Theory of Acceptance and Use of Technology 2.

## Discussion

### Principal Findings

This study explored the experiences and acceptance of community-based mHealth services among residents in an underserved rural area in South Korea, revealing that these services were gradually integrated into participants’ everyday lives. Through regular monitoring, participants reported that they could clearly sense changes in their bodies, that they were no longer managing their health alone, and that using the devices had ultimately become a part of their daily routine. Nurses working in the public health centers provided continuous support, such as troubleshooting and accountable monitoring, which facilitated participants’ adoption of the device. Interpreted through the UTAUT2 framework, these experiences indicate that performance expectancy, social influence, facilitating conditions, and habit jointly reinforced behavioral intention and continued use of community mHealth services among the research participants.

### Performance Expectancy and Perceived Health Benefits

Performance expectancy is a central predictor of technology adoption and continued use within the UTAUT2 framework [20]. The findings of this study further support this construct, as participants emphasized perceived health benefits as the primary motivation for engaging with mHealth services. Real-time visualization of vital signs and the results of physical activity allowed participants to link their daily behaviors to measurable health indicators. They described specific lifestyle modifications, such as reducing carbohydrate intake, increasing vegetable consumption, and maintaining step-count targets, which they attributed directly to feedback from the devices and apps. Some participants reported perceived prevention of disease progression. For example, they detected prediabetes earlier than they would have through routine clinic visits. These experiences align with prior mHealth research showing that digital self-monitoring has been associated with improvements in chronic disease management and prevention outcomes, such as blood pressure and glycemic control, especially when combined with coaching or tailored feedback [28-30]. Studies using the UTAUT2 framework in older adult and chronic illness populations have similarly reported that strong beliefs about health improvement are among the most powerful determinants of intention to use and actual usage behavior. In this study, these beliefs were reinforced as participants could literally “see” their improvements reflected in the app’s numbers and graphs, allowing them to connect these visualized changes to concrete behavioral adjustments [24].

Our findings extend this literature by illustrating how performance expectancy operates in a rural public health setting where clinic visits are infrequent and travel distances are long. For many participants, mHealth services effectively compressed the monitoring interval from every few months in a hospital to daily at home. This shift appears to have strengthened the perceived use of the technology, as participants came to view mHealth as a useful tool for maintaining stability in their chronic conditions between formal clinic visits [19,24].

### Role of Nurses, Social Influence, and Hedonic Motivation

Our findings highlight the pivotal role of nurses and social relationships in shaping both social influence and facilitating conditions. In this setting, community health center nurses delivered hands-on training, offered ongoing troubleshooting, interpreted health data, and coordinated group exercise and peer interactions. These human-in-the-loop functions enabled older adults and individuals with low digital literacy to overcome early usability barriers and develop trust in mHealth systems, consistent with prior evidence on nurse-led telehealth and digital chronic care support [2,3,5].

Nurses played dual roles within the UTAUT2 framework. They functioned as facilitating conditions by providing training, troubleshooting, and device maintenance, while simultaneously serving as sources of social influence through encouragement, accountability, and trust. This overlap highlights the limitations of rigid construct boundaries in team-based primary care contexts.

As group activities and peer comparison increased, social influence shifted from individual encouragement to a shared sense of “doing it together.” This collective engagement enhanced hedonic motivation [11,30]. Participants described enjoyment, friendly competition, and pride in meeting daily goals. Elements such as comparing step counts or receiving recognition during group sessions introduced a game-like dynamic that strengthened adherence. Although small incentives were appreciated, many participants emphasized intrinsic motivation (“staying healthy”) over external rewards. As a result, device use was not perceived as burdensome [17].

Overall, these findings, which are consistent with evaluations of mHealth use among middle-aged Korean adults in their 50s and 60s [24], suggest that the successful scale-up of digital health interventions in rural areas depends more on relationship-centered care models than on technology dissemination alone. At the study sites, nurses were the only health care professionals available to manage the mHealth services, as recruiting physical therapists, nutritionists, or other allied health professionals was particularly challenging in these rural settings. Because the study sites were public health centers, nurses naturally assumed human-in-the-loop roles, providing continuous monitoring, encouragement, and contextualized feedback to participants. However, as primary care increasingly emphasizes high-quality, team-based care, long-term strategies are needed to establish mobile and multidisciplinary primary care teams, rather than relying solely on nurses, in order to effectively support chronic disease management among rural residents [31].

Furthermore, implementation science research is needed to better understand how mHealth interventions are integrated into routine clinical practice, sustained over time, and adapted to the contextual constraints of underserved rural settings. In addition, economic factors, including program financing, workforce costs, and patients’ willingness to pay, remain relatively understudied and should be systematically examined to inform the scalability and sustainability of rural digital health initiatives.

### Habit Formation and Long-Term Engagement

Habit was a key construct shaping participants’ sustained engagement with mHealth services. Most participants reported that checking their blood pressure, reviewing their step counts, or looking at daily summaries had become automatic behaviors, often compared to brushing their teeth. These routines were anchored at specific times, on waking, after exercise, and before going to bed, and were reinforced by consistent cues such as device placement, app notifications, and scheduled group activities. This pattern aligns with recent technology acceptance research emphasizing that, beyond initial intention, habit and automaticity are critical for explaining long-term use of mHealth and wearable technologies among older adults and people with chronic diseases [24].

Designing interventions that incorporate clear goals, progress visualization, social reinforcement, and modest gamification may help accelerate the transition from conscious effort to self-sustaining habit. Importantly, the sources of enjoyment described here were not purely digital; they were relational and communal, arising from doing activities with peers and being acknowledged by nurses and staff.

### Rural Digital Divide, Usability Barriers, and Inclusive Design

Despite generally positive experiences, older participants and those with minimal prior digital exposure reported usability challenges. These participants often found initial setup confusing, required 6 months or longer to feel comfortable with the devices, and remained more dependent on nurses or family members for troubleshooting. Difficulties included small text, complex menus, unreliable Bluetooth connections, and anxiety about “breaking” the device through incorrect use. These findings echo previous findings that older adults in rural settings tended to have lower levels of education, high rates of frailty, and limited prior exposure to smartphones and apps constrain digital health adoption [32]. In terms of the reminder system, using color-based alerts or a 5-star rating method instead of text-based warning messages may need to be considered. Nevertheless, our findings demonstrate that these participants were able to develop confidence and proficiency over time when supported by consistent hands-on guidance from health care staff or coordinators. Although their adaptation period was longer, participants eventually became familiar with the devices and expressed a sense of empowerment in being able to use new technology, which in turn enabled them to experience the benefits of improved self-management and health outcomes. These results underscore the need for structured, hands-on guidance materials and training protocols tailored specifically for older adults in rural communities, ensuring that digital health innovations remain accessible and equitable for populations with lower digital readiness.

### Limitations

Although this study provides valuable insights into older adults’ experiences with mHealth for chronic disease prevention and management in rural Korea, several limitations should be noted. Participants were current users with at least 6 months of engagement, which may overrepresent positive experiences and undercapture reasons for nonadoption or discontinuation. Former users were not included. Social desirability bias may have influenced self-reported technology use and perceived value. In addition, the study was conducted within a specific rural public health context, and findings should be interpreted in light of local health care structures. Finally, detailed usage patterns and clinical outcomes associated with the digital health devices were not examined and warrant further investigation.

### Conclusions

In this mixed methods study conducted in an underserved rural area of Korea, community-based mHealth services were successfully integrated into the daily lives of residents living with, or at risk for, chronic conditions. Mobile monitoring devices, supported by public health center nurses and peer networks, enabled participants to incorporate self-monitoring into their everyday routines.

Guided by the UTAUT2 framework, the findings identified performance expectancy, social influence, facilitating conditions, and habit as the most salient determinants of technology acceptance and continued use. Participants described perceiving tangible bodily changes, experiencing reduced isolation in managing chronic conditions, and relying on nurses as supportive “human-in-the-loop” partners operating in the background. Over time, these experiences helped participants establish stable and sustainable self-monitoring behaviors.

These results suggest that digital health strategies for rural and aging communities should move beyond simple device deployment. Instead, such strategies should invest in intuitive system design, hands-on onboarding, and ongoing nurse-involved technical and interpretive support. In addition, intentionally fostering group-based activities and peer connections may strengthen social influence and hedonic motivation. Finally, stable funding and sustained organizational commitment will be essential to maintaining trust, reducing digital inequality, and realizing the long-term benefits of remote monitoring–supported self-management in underserved rural populations.

## Supplementary material

10.2196/91368 Multimedia Appendix 1 Unified Theory of Acceptance and Use of Technology 2-based codebook derived from directed content analysis.

## Abbreviations

AI: artificial intelligence
mHealth: mobile health
UTAUT2: The Unified Theory of Acceptance and Use of Technology 2
