# Supplementary material for: Experiences and Acceptance of Community-Based Mobile Health Services Among People in Underserved Rural Areas of Korea: Mixed Methods Study
Source: JMIR Form Res. 2026 Jun 22;10:e91368. doi: 10.2196/91368 (PMC13286076; doi:10.2196/91368)
Supplement: Multimedia Appendix 1 [file formative-v10-e91368-s001.docx]

**Table S1.** UTAUT2-based codebook derived from directed content analysis.

| UTAUT2^a^ Construct | code | Code description |
| --- | --- | --- |
| Performance expectancy | - Data-driven health awareness | - Awareness and understanding of health status through real-time monitoring |
|  | - Behavior activation | - Motivation to engage in self-management behaviors |
|  | - Perceived health benefits | - Perceived improvements in health outcomes and prevention of disease progression |
| Effort expectancy | - Initial usability barrier | - Difficulties experienced during early stages of learning and using the technology |
|  | - Gradual adaptation | - Progressive improvement in usability through repeated use and experience over time |
|  | - Preference for simplicity | - Preference for simple and easy-to-use functions that minimize cognitive effort |
|  | - Visual accessibility | - Preference for clear visual design elements such as large text, icons, and intuitive layouts |
| Social influence | - Family support | - Encouragement and assistance from family members that facilitate technology use |
|  | - Peer influence | - Motivation derived from observing or interacting with peers using the technology |
|  | - Provider recommendation | - Influence of healthcare providers’ advice or endorsement on technology adoption and use |
| Facilitating conditions | - Technical support availability | - Availability of assistance for troubleshooting technical issues and device malfunctions |
|  | - Supported learning | - Development of user proficiency through repeated training and hands-on guidance |
|  | - Continuous guidance | - Ongoing support and follow-up provided by healthcare staff to sustain engagement |
|  | - Human-mediated system | - Integration of human support with digital technology to enhance usability and trust |
| Habit | - Routine embedding | - Routine embedding \| Integration of technology use into daily routines and habitual practices |
|  | - Automaticity | - Use of technology with minimal conscious effort due to repeated practice |
|  | - Cue-triggered action | - Technology use prompted by environmental or contextual cues |
| Hedonic Motivation | - Enjoyment of monitoring | - Pleasure derived from tracking and reviewing personal health data |
|  | - Achievement satisfaction | - Sense of accomplishment from meeting health-related goals |
|  | - Intrinsic motivation | - Engagement driven by internal values such as maintaining or improving health |

^a^UTAUT2: Unified Theory of Acceptance and Use of Technology 2
